# Transforming Health and Resiliency Through Integration of Values-based Experiences: Implementation of an Electronic Evidence-based Whole Health Clinical Program

**DOI:** 10.2196/26030

**Published:** 2021-06-29

**Authors:** Jolie N Haun, Jacquelyn Paykel, Christine Melillo

**Affiliations:** 1 Research Service James A. Haley Veterans' Hospital Tampa, FL United States; 2 Department of Community & Family Health University of South Florida Tampa, FL United States; 3 Whole Health Service James A. Haley Veterans' Hospital Tampa, FL United States

**Keywords:** virtual care, group medical appointment, complementary and integrative health, veteran, implementation

## Abstract

**Background:**

Complementary and integrative health (CIH) is the foundation of the Department of Veterans Affairs (VA) Whole Health System program (WH), including Transforming Health and Resiliency through Integration of Values-based Experiences (THRIVE). The global COVID-19 pandemic prompted an urgent need to provide services such as THRIVE while following guidelines for social distancing.

**Objective:**

The objective of this paper was to describe the systematic implementation of THRIVE using an electronic delivery model.

**Methods:**

The study involved an observational clinical program implementation project using the RE-AIM framework to contextualize the implementation strategies and results, and then the implementation of an electronically delivered CIH group medical appointment program (eTHRIVE).

**Results:**

Clinical staff transitioned to 100% electronic delivery of the THRIVE curriculum using the new eTHRIVE delivery model. The current electronic delivery model, eTHRIVE, has effectively enrolled 10-12 veterans per cohort, with 8 cohorts, totaling 87 veterans to date. eTHRIVE attrition has been 6% (5/87) since initiation.

**Conclusions:**

The current climate of the VA WH programmatic initiative combined with the public health needs during a global pandemic prompted the move of THRIVE program into an electronic format to broaden scalability and reach.

## Introduction

Opioid reduction, mental health management, and suicide prevention are named priorities for the Department of Veterans Affairs (VA) and the nation. Chronic pain conditions are often not responsive to pharmacological treatment long term [1], yet opioid agents have been the mainstay of treatment. Mental health conditions are often complex, requiring difficult work from patients and are responsive to an integrated approach of medication, lifestyle, and psychological counseling when patients complete treatment programs [2-5]. The urgent need for improved pain and mental health management coupled with veteran demand resulted in the VA Complementary and Integrative Health (CIH) mandate in Subtitle C of the Comprehensive Addiction and Recovery Act (CARA) [6]. In a strategic responsive effort, the VA has implemented a systematic effort to promote patient-centered care through the implementation of a Whole Health System program (WH) of care [7].

The WH integrates allopathic and CIH care, focusing on patients’ goals and priorities, with peer-led support, personalized health planning, WH coaches, and well-being classes. There are 3 major components of the WH: (1) the pathway, (2) well-being programs, and (3) whole health clinical care. The pathway introduces veterans to the concepts of WH and helps them identify their health goals and develop their personal health plan. Through the pathway, veterans are introduced to the components of Proactive Health & Well-Being Programs to support their personal health, including health coaching and CIH services, such as acupuncture, chiropractic care, meditation, therapeutic massage, biofeedback, clinical hypnosis, guided imagery, yoga, and Tai Chi. Finally, the third and final component is whole health clinical care, which is a clinical care model that includes use of a WH paradigm for providing a higher level of care in both allopathic and CIH settings, delivered by a multidisciplinary team of clinical providers (eg, physician, social worker, psychologist, pharmacist). Although this third component is critical for combining the delivery of CIH and clinical care, a standardized evidence-based program has not yet been adopted by VA. Standardization of content and delivery of a program supports broader program fidelity efforts (ie, evaluation, treatment effectiveness research, and service administration) [8]. Basing clinical programs on evidence in published literature assures program effectiveness for targeted outcomes (ie, informed clinical decisions, patient outcomes) [9]. Without standardized evidence-based clinical programs, health care programs may stagnate to the status quo, providers and facilities cannot anticipate fidelity, and veterans cannot all access the same quality programs across facilities. There is a need for a standardized evidence-based WH Proactive Health & Well-Being Programs. In addition, group medical appointments provide a sustainable model, to meet the demand of the Proactive Health & Well-Being Programs.

*Transforming Health and Resiliency through Integration of Values-based Experiences*, more commonly known as THRIVE, based on evidence-based principles of whole health/integrative medicine [10-12], positive psychology [13,14], and acceptance and commitment therapy [15,16], is a clinical program to improve access to CIH modalities for chronic pain, mental health, and suicide prevention services. THRIVE has been implemented in multiple VA regions and is gaining attention as a potential opportunity to fill the need for the WH Proactive Health & Well-Being Programs component. THRIVE is a 14-week curriculum-based group medical appointment for veterans, facilitated by an interdisciplinary team of clinicians. Each weekly group medical appointment focuses on a different component of wellness (eg, financial, spiritual, physical) and is led by a knowledgeable clinician (eg, social worker, chaplain, physical therapist). THRIVE is currently the only evidence-based clinical program in practice that fulfills the 3 components of WH, namely, the pathway, well-being programs, and clinical care, and is identified a best practice by the VA Office of Patient Centered Care and Cultural Transformation (OPCCCT). Although this program was developed for face-to-face delivery, it was adapted for the remote electronic delivery format during the COVID pandemic due to the suspension of face-to-face group medical appointments.

The purpose of this paper is to describe the systematic, rapid implementation of THRIVE using an electronic delivery model. Our intention is to provide a practical example that may be applied to other CIH and group medical appointments as they transition to electronic delivery while maintaining integrity and quality. Evaluation of this implementation is ongoing.

## Methods

### Overview

RE-AIM is an ideal evidence-based framework for contextualizing the implementation of THRIVE [17]. RE-AIM’s 5 elements (*reach the target population, effectiveness, adoption, implementation,* and *maintenance*) provide a systematic framework for planning and implementing programs from the initial efforts of reaching audiences, to establishing effectiveness, then on to promoting adoption, implementation, and ultimately sustaining maintenance over time [18-20]. It is also noteworthy that RE-AIM is a commonly used framework for implementing evidence-based programs in large health care systems, such as the VA, making it a popular and practical framework for such implementation efforts as the THRIVE program. The RE-AIM model has been the guiding framework throughout the development and implementation of THRIVE; however, the pandemic required a real-time adaptation that was expeditious in nature. Through an ongoing development and quality improvement between the clinical team and the implementation team, RE-AIM has been used to provide a framework to contextualize and guide eTHRIVE implementation. Planning and implementation of THRIVE are described using the 5 elements.

### Reach the Target Population

THRIVE was developed by a female veteran physician for female veterans. The program was provided within VA women’s health care clinics, using a clinician-informed referral process to ensure the program was aligned and appropriate for the *target population* (female veterans with chronic conditions). Subsequently, based on patient outcomes and feedback, the THRIVE program was expanded and modified for male veterans. The program moved from women’s health to the larger WH service to ensure access and integration throughout the health care system to reach all veterans in need of access to CIH modalities in managing not only chronic conditions but also living a values-based life.

### Effectiveness

Effectiveness can be measured in many ways (eg, clinical outcomes, organizational outcomes). A recent evaluation of 201 female veteran participants in THRIVE demonstrated the *effectiveness* of this program at improving self-reported mental health outcomes, such as depression, anxiety, psychological inflexibility, and experiential avoidance, as well as improvements in life satisfaction and work pain interference [21]. Organizational outcomes such as cost-effectiveness are currently under review.

### Adoption

An organizational cultural transformation is imperative for adoption of innovative programs such as THRIVE [7,22,23]. THRIVE gained popular support and *adoption* by clinical teams, administration, and veterans. Once adopted at the originating veterans’ hospital, implementation efforts expanded to facilities within the Veterans Integrated Service Network (VISN) followed by spread to neighboring VISNs.

### Implementation and Maintenance

The VA is an established leader in implementation science. The *implementation* of eTHRIVE was subsequent to many implementation efforts to the original THRIVE program. Implementation strategies [24] facilitated the strategic steps used to reach the population (ie, veterans) with an effective program (ie, THRIVE) through a new service delivery (ie, electronic). This rapid practical application of implementation strategies was based on best practices [24], literature [24], experience of the implementation team [25,26], and the needs of the program in the real-time implementation. [*Implementation strategies are hereafter presented in parenthesis in the following sections.]*

## Results

### eTHRIVE Overview

The eTHRIVE process resulted in 14 strategic steps and implementation strategies, and data on program uptake and attrition. In its initial development, to support fidelity, scalability, and maintenance of THRIVE, an implementation manual and a train-the-trainer program (*use train-the-trainer strategies, make training dynamic*), THRIVE Immersion, were developed for VA staff to facilitate and sustain the THRIVE program within various clinical settings.

As THRIVE moved into its fourth year of implementation in 2020, a growing waitlist and limited resources posed challenges for access and hopes for establishing the scalability of this clinical program. As with most programs, staffing, space, and other resources that are often in high demand illustrate the limitations of scaling up access to the program. Transferring the earlier in-person THRIVE program to a standardized electronic format for delivery creates an exponential opportunity for scalability, mobility, and control. For example, in the face-to-face delivery model, 10 veterans per cohort, averaging 13 cohorts per year, totaled 400 veterans to date. Whereas with an electronic version (*stage implementation scale up*) processes allow approximately 10 veterans per cohort (to maintain a small, yet high-quality dynamic group), with 50 cohorts per year, resulting in 500 veterans served per year, exponentially increasing the number of veterans served annually. The eTHRIVE program not only creates an opportunity to broaden reach through scalability, but also presents benefit through resource conservation, cutting costs in materials, and space. For example, some of the in-person materials were modified to electronic materials (eg, presentations converted to video), thus eliminating the need for staff to be present for those segments of the program, creating more time to serve additional cohorts. It is critical to understand there is an issue with supply and demand when considering meeting space within most medical centers. With competing interests and high demand, remote delivery becomes the most economical strategy to broaden reach to veterans. Although the program creator and early adopting providers saw the potential of delivering the program as an electronically delivered service, VA policies and infrastructure did not support the opportunity for eTHRIVE in the current system. However, recent COVID-19 pandemic events have thrust the VA into a new era of policy and opportunity for innovating in the delivery of care using electronic resources while reducing contact. As such, providers must now deliver services virtually, including WH care. This presents a timely opportunity for implementing the THRIVE program in the broader VA system.

### Implementation and Maintenance of eTHRIVE

#### Overview of Steps

**Figure 1:**
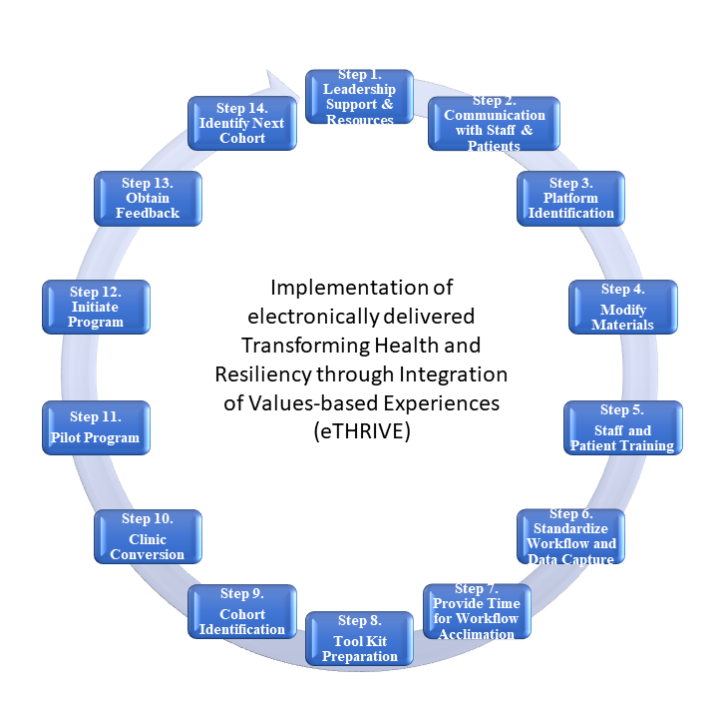
14-step process for implementation of electronically delivered Transforming Health and Resiliency through Integration of Values-based Experiences (eTHRIVE).

Implementation of eTHRIVE was a clinical care process that required clear communication and quick response to ever evolving public health pandemic crisis. During this unprecedented public health emergency, WH providers, staff, and clients acted together to support continuity of care through electronically delivered services. The 14-step process is shown in Figure 1.

#### Step 1

The first step was to garner leadership support (involve executive boards) and resources (hardware, software, ongoing technical support as staff learned new systems, reallocation of funding from salaries to equipment) for the transfer and implementation of eTHRIVE. This critical step involved establishing stakeholder buy-in and justification of necessary resources to transition to electronic delivery (*change service sites*) of THRIVE in the wake of a larger transition required to maintain continuity and access to care in the wake of establishing new paradigms of remote care in the context of a global pandemic. Staff were organized into small groups (*organize clinician implementation team meetings*) for brain storming implementation (*conduct local consensus discussions*).

#### Step 2

Communication of transition with staff and veteran patients was imperative. As in-person group medical appointments came to an immediate halt at the onset of the COVID-19 pandemic, the disruption of workflow for staff and potential discontinuation of services to veteran patients presented problems that required immediate attention. As such, communication about the transition of THRIVE to electronic delivery (eTHRIVE) was prioritized as a critical need to establish new workflow patterns and ensure veteran patients continued to have access to care (*promote adaptability*).

#### Step 3

The THRIVE team leveraged existing resources to identify a platform (*tailor strategies, promote adaptability*) to provide electronically delivered services. The platform needed to be accessible to all levels of technology-proficient providers, staff, and clients. Intuitive platforms were most desirable to facilitate platform training (*facilitation*) to all who would access the platform. The platform also needed to include an educational package such as whiteboards, polling, and shared video viewing. After reviewing the locally available platforms, an appropriate platform was selected.

#### Step 4

eTHRIVE materials were reviewed to determine if they were already in the appropriate electronic format (ie, video, PowerPoint) or if they needed to be revised (*audit and provide feedback*) to an optimal presentation mode for the new platform. Paper-based materials would be delivered in the eTHRIVE tool kit (see Step 5).

#### Step 5

It was critical to prepare staff with skills and proficiency in using technology to deliver eTHRIVE [in addition to the THRIVE immersion training (train-the-trainer training)]. Most clinic staff worked within the clinic weekdays during regular business hours. They had no need for computer, video, or audio equipment to be assigned to them for use from home. This caused a delay in standing up the eTHRIVE program. Lack of equipment and software for work from home created transition stress for staff and providers. Once individuals had the technical mobility for remote work (*change physical structure and equipment*), training on the new platform began. We ran through scenarios, tested platform functions, and delegated “champions” (*identify and prepare champions, provide local technical assistance*) who could be a resource for technical issues. These champions (*identify early adopters*) were instrumental in assisting team members with transitions to the home environment. As staff and providers became familiar with the platform, we began training patients. With each patient training, staff became more familiar with the platform and were able to independently modify trainings (*conduct ongoing training*) to best meet patient needs. Teams met routinely with staff, consultants, and leadership to provide ongoing consultation (*provide ongoing consultation*) and continued education (*conduct educational meetings*).

#### Step 6

While staff and clients became familiar with the platform and new processes, eTHRIVE leadership, in collaboration with researchers (*use data experts, use an implementation advisor*), identified and developed standardized tools (*develop and implement tools for quality monitoring*) and processes (*develop and organize quality monitoring systems*) to capture patient and workflow data. Data, collected in an organized, standard way across providers and time, was used to identify glitches in the new system. These issues were brought to the attention of leadership (*inform local opinion leaders*) and addressed in a timely fashion to maintain continuity of care, even though this was such a large transition.

#### Step 7

When transitioning into a remote work environment, it is critical to give staff time and support to acclimate to their new workflow dynamics. WH leadership recognized the stress of these transitions and built time into plans to allow acclamation to this new work environment and all the changes that come with a public health emergency requiring stay at home orders. Most urgent transitions can be burdensome, creating stress not only among clients but also for the staff and providers who provide care. Stay at home orders meant many staff and providers were also juggling personal life demands with new work protocols while finding space and equipment in the home for all household members to continue addressing their responsibilities.

#### Step 8

The next step was to create eTHRIVE tool kits (*develop educational materials*). Each tool kit contained a THRIVE workbook, THRIVE journal, art supplies, markers, dry erase response paddle, and other supplies used with each of the 14 units. Tool kits were mailed to participants (*distribute educational materials*) in a single package. Materials for each unit were placed in individual envelopes. The tool kit also included additional worksheets, metrics, and packets for clients to complete in specific weeks with a self-addressed stamped envelope to return metrics to the WH team.

#### Step 9

Although identification of eTHRIVE cohorts was a step in this process, the means for identification did not change from identification of THRIVE cohorts. Each cohort is generated from consults. Consults were typically entered into the VA electronic health record by primary care providers familiar with WH and the benefits of group medical appointments such as THRIVE. WH staff contacted veterans with pending consults and invited them to participate in eTHRIVE. When clients accepted the invitation, staff further verified acceptability and capability with electronically delivered care (*intervene with patients/consumers to enhance uptake and adherence, prepare patients/consumers to be active participants*).

#### Step 10

Conversion of clinics (*alter incentive/allowance structures, change record systems*) from standard to virtual care was conducted to get organizational virtual care credit (*make billing easier*). This conversation required rebuilding every clinic, creating a grid and renaming these clinics, and obtaining time-consuming administration approvals.

#### Step 11

Pilot an eTHRIVE group medical appointment with veterans (*assess for readiness and identify barriers and facilitators*) to ensure usability. Piloting the eTHRIVE group was an essential component to ensuring the staff and veterans were comfortable using the technology, as well as ensuring the technology and delivery modes were fully functional for eTHRIVE delivery.

#### Step 12

Conduct an eTHRIVE program with veteran cohorts. Once all systems, processes, and materials were prepared and piloted, the eTHRIVE program was conducted as a fully functioning electronically delivered group medical appointment.

#### Step 13

As in any implementation of a large-scale process change, rapid feedback on the process (*conduct cyclical small tests of change*) is imperative and a critical aspect of maintaining optimal implementation over time. Hard copy standard metrics were sent to all participants (N=87) with the majority (n=69) returned to the team by postal mail. There is typically a response burden when participants are required to mail (postal) paper data to clinicians or evaluators. Electronic data collection was subsequently implemented to be in alignment with delivery and remote accessibility. Attrition (5/87, 6%) and acceptability were tracked (*purposely reexamine the implementation*) as a marker for the new platform. Additional feedback measures included feedback surveys (*obtain and use patients/consumers and family feedback*) after each episode of care and recap after each session with staff to improve performance. These data (ie, acceptability, feedback surveys) are currently being analyzed.

#### Step 14

Identify next cohort and repeat delivery of eTHRIVE with integration of feedback and lessons learned to enhance delivery support program implementation maintenance over time. As with all program implementation and evaluation, eTHRIVE used this approach with the first groups to elicit feedback (*involve patients/consumers and family members*) and inform revisions (*capture and share local knowledge*) to improve the program process and delivery to optimize veterans’ experience and promote program outcomes.

### Uptake and Attrition

Clinical staff have transitioned to 100% electronic delivery of the THRIVE curriculum using the new eTHRIVE delivery model. The current electronic delivery model, eTHRIVE, has effectively enrolled 10-12 veterans per cohort, with 8 cohorts, totaling 87 veterans to date. eTHRIVE attrition has been 6% (5/87) since initiation. As efforts move forward, program content will be fully re-designed for electronic format and program administrators will leverage opportunities, such as the use of tablets, while balancing barriers associated with those same opportunities, including acquisitions.

## Discussion

### Principal Findings

The Whole Health Program is the VA’s systematic response to provide a CIH care approach to help veterans manage their health, particularly mental health and pain conditions associated with opioid use and suicide. THRIVE, an evidence-based clinical CIH program, has been systematically transitioned to an electronic delivery model to ensure continuity and access to care in light of the COVID-19 crisis, and the resultant critical need for remote access of care.

Implementation of eTHRIVE was a clinical care process that transitioned face-to-face group medical appointments to electronic delivery group medical appointments in 14 systematic steps. From garnering stakeholder support and selecting a virtual platform, to implementing, evaluating, and refining the program, the process was systematically conducted to ensure successful delivery of eTHRIVE for targeted veteran patients in need.

eTHRIVE presents a multitude of opportunities, including moving to a more pliable applicable platform that is sustainable and remotely accessible, improving reach to marginalized populations. However, eTHRIVE implementation also presented a multitude of potential challenges. First, government-issued equipment for speed and security is required, and patients require technology such as smart pads and personal computers. In addition to equipment and bandwidth requirements, information technology support, which is often limited, is a critical resource required for implementation. Besides, adequate transition time is required to get staff and patients prepared for electronic delivery. While the challenges and resource limitations pose barriers for any electronically delivered program implementation, the benefits of remote access and patient outcomes associated with CIH in a group medical appointment setting warrant effort and resources.

Because of the urgency for continuity of care during the COVID-19 pandemic, the transition and implementation of THRIVE to eTHRIVE was done with urgency. Some limitations to this rapid implementation include lack of a needs and readiness assessment and co-creation of a stakeholder-invested implementation plan, and solutions (eg, adequate equipment, employee telework protocols, and IT support) had to be developed concurrently and reactively opposed to a proactive strategic process. Although these limitations clearly present challenges to the implementation process, the real-world implementation necessitated by a global pandemic presented a unique opportunity to create a remote delivery option that was previously prohibitive, due to outdated confidentiality and privacy policies that did not reflect modern technological capabilities in an emerging era of remotely delivered health care.

### Conclusions

This paper shows the value of outlining the strategic process for adapting a shared medical appointment approach to a remote delivery model while maintaining the integrity of the group medical experience. The current climate of the VA WH programmatic initiative combined with the health care remote access needs due to the current pandemic creates an opportune time to move group medical appointment clinical care programs into an electronic format to broaden scalability and reach. The VA now has the motivation—and need—to leverage infrastructure and resources to implement group medical appointment clinical care programs such as eTHRIVE. The transition from face-to-face to electronic delivery of eTHRIVE occurred in 14 pseudo-simultaneous steps. As implementation of this exemplar group medical appointment clinical care program advances, dissemination of data findings, return on investment, cost-effectiveness, and factors effecting electronic implementation will be critical in moving the science forward as the VA strategizes the electronic delivery of care to all veterans while reducing contact.

## Abbreviations

CARA: Comprehensive Addiction and Recovery Act
CIH: complementary and integrative health
OPCCCT: Office of Patient Centered Care and Cultural Transformation
THRIVE: Transforming Health and Resiliency through Integration of Values-based Experiences
VA: Department of Veterans Affairs
VISN: Veterans Integrated Service Network
WH: Whole Health System program

## References

[1] Chou R, Turner JA, Devine EB, Hansen RN, Sullivan SD, Blazina I, Dana T, Bougatsos C, Deyo RA (2015). The effectiveness and risks of long-term opioid therapy for chronic pain: a systematic review for a National Institutes of Health Pathways to Prevention Workshop. Ann Intern Med.

[2] Nemeroff CB (2020). The State of Our Understanding of the Pathophysiology and Optimal Treatment of Depression: Glass Half Full or Half Empty?. Am J Psychiatry.

[3] Zhou Y, Sun L, Wang Y, Wu L, Sun Z, Zhang F, Liu W (2020). Developments of prolonged exposure in treatment effect of post-traumatic stress disorder and controlling dropout rate: A meta-analytic review. Clin Psychol Psychother.

[4] Spidel A, Lecomte T, Kealy D, Daigneault I (2018). Acceptance and commitment therapy for psychosis and trauma: Improvement in psychiatric symptoms, emotion regulation, and treatment compliance following a brief group intervention. Psychol Psychother.

[5] Krystal JH, Davis LL, Neylan TC, A Raskind Murray, Schnurr PP, Stein MB, Vessicchio J, Shiner B, Gleason TD, Huang GD (2017). It Is Time to Address the Crisis in the Pharmacotherapy of Posttraumatic Stress Disorder: A Consensus Statement of the PTSD Psychopharmacology Working Group. Biol Psychiatry.

[6] (2017). VHA Directive 1137: Provision of Complementary and Integrative Health (CIH). U.S. Department of Veterans Affairs.

[7] Abadi Melissa H, Barker Anna M, Rao Sowmya R, Orner Michelle, Rychener David, Bokhour Barbara G (2021). Examining the Impact of a Peer-Led Group Program for Veteran Engagement and Well-Being. J Altern Complement Med.

[8] Mowbray CT, Holter MC, Teague GB, Bybee D (2016). Fidelity Criteria: Development, Measurement, and Validation. American Journal of Evaluation.

[9] Fineout-Overholt E, Melnyk B, Schultz A (2005). Transforming health care from the inside out: advancing evidence-based practice in the 21st century. J Prof Nurs.

[10] Yang NY, Wolever RQ, Roberts R, Perlman A, Dolor RJ, Abrams DI, Ginsburg GS, Simmons LA (2017). Integrative health care services utilization as a function of body mass index: A BraveNet practice-based research network study. Advances in Integrative Medicine.

[11] Fletcher CE, Mitchinson AR, Trumble EL, Hinshaw DB, Dusek JA (2016). Perceptions of other integrative health therapies by Veterans with pain who are receiving massage. J Rehabil Res Dev.

[12] Crocker RL, Grizzle AJ, Hurwitz JT, Rehfeld RA, Abraham I, Horwitz R, Weil A, Maizes V (2017). Integrative medicine primary care: assessing the practice model through patients' experiences. BMC Complement Altern Med.

[13] Seligman MEP, Fowler RD (2011). Comprehensive Soldier Fitness and the future of psychology. Am Psychol.

[14] Resnick SG, Rosenheck RA (2006). Recovery and positive psychology: parallel themes and potential synergies. Psychiatr Serv.

[15] Lang Ariel J, Schnurr Paula P, Jain Sonia, He Feng, Walser Robyn D, Bolton Elisa, Benedek David M, Norman Sonya B, Sylvers Patrick, Flashman Laura, Strauss Jennifer, Raman Rema, Chard Kathleen M (2017). Randomized controlled trial of acceptance and commitment therapy for distress and impairment in OEF/OIF/OND veterans. Psychol Trauma.

[16] Forman EM, Herbert JD, Moitra E, Yeomans PD, Geller PA (2007). A randomized controlled effectiveness trial of acceptance and commitment therapy and cognitive therapy for anxiety and depression. Behav Modif.

[17] Glasgow R, Harden S, Gaglio B, Rabin Borsika, Smith Matthew Lee, Porter Gwenndolyn C, Ory Marcia G, Estabrooks Paul A (2019). RE-AIM Planning and Evaluation Framework: Adapting to New Science and Practice With a 20-Year Review. Front Public Health.

[18] Glasgow RE, Dickinson P, Fisher L, Christiansen S, Toobert DJ, Bender BG, Dickinson LM, Jortberg B, Estabrooks PA (2011). Use of RE-AIM to develop a multi-media facilitation tool for the patient-centered medical home. Implement Sci.

[19] Gaglio B, Shoup JA, Glasgow RE (2013). The RE-AIM framework: a systematic review of use over time. Am J Public Health.

[20] Bennett GG, Glasgow RE (2009). The delivery of public health interventions via the Internet: actualizing their potential. Annu Rev Public Health.

[21] Haun JN, Paykel J, Alman AC, Patel N, Melillo C (2020). A complementary and integrative health group-based program pilot demonstrates positive health outcomes with female Veterans. Explore (NY).

[22] Taylor SL, Bolton R, Huynh A, Dvorin K, Elwy AR, Bokhour BG, Whitehead A, Kligler B (2019). What Should Health Care Systems Consider When Implementing Complementary and Integrative Health: Lessons from Veterans Health Administration. J Altern Complement Med.

[23] Bokhour BG, Fix GM, Mueller NM, Barker AM, Lavela SL, Hill JN, Solomon JL, Lukas CV (2018). How can healthcare organizations implement patient-centered care? Examining a large-scale cultural transformation. BMC Health Serv Res.

[24] Powell BJ, Waltz TJ, Chinman MJ, Damschroder LJ, Smith JL, Matthieu MM, Proctor EK, Kirchner JE (2015). A refined compilation of implementation strategies: results from the Expert Recommendations for Implementing Change (ERIC) project. Implement Sci.

[25] Haun J, Chavez M, Hathaway W, Antinori N, Melillo C, Cotner BA, McMahon-Grenz J, Zilka B, Patel-Teague S, Messina W, Nazi K (2018). Virtual Medical Modality Implementation Strategies for Patient-Aligned Care Teams to Promote Veteran-Centered Care: Protocol for a Mixed-Methods Study. JMIR Res Protoc.

[26] Haun J (2018). Advancing Implementation Science in Healthcare. https://www.eventscribe.com/2018/ACRM/fsPopup.asp?Mode=presInfo&PresentationID=401779.

